# Role of hypoxia-inducible factor-1α and CD146 in epidermal growth factor receptor-mediated angiogenesis in salivary gland adenoid cystic carcinoma

**DOI:** 10.3892/mmr.2015.3815

**Published:** 2015-05-22

**Authors:** WEI-MING WANG, ZHI-LI ZHAO, WEN-FENG ZHANG, YI-FANG ZHAO, LU ZHANG, ZHI-JUN SUN

**Affiliations:** 1 The State Key Laboratory Breeding Base of Basic Science of Stomatology and Key Laboratory of Oral Biomedicine Ministry of Education, Wuhan University, Wuhan, Hubei 430079, P.R. China; 2 Department of Oral and Maxillofacial-Head and Neck Oncology School and Hospital of Stomatology, Wuhan University, Wuhan, Hubei 430079, P.R. China

**Keywords:** adenoid cystic carcinoma, tissue microarray, epidermal growth factor receptor, hypoxia-inducible factor-1α, angiogenesis, CD146

## Abstract

Adenoid cystic carcinoma (AdCC) of the salivary gland in the head and neck is characterized by indolent yet persistent growth, multiple local recurrences and early hematogenous metastasis. Considering the possible association between the epidermal growth factor receptor (EGFR) signaling pathway and angiogenesis in various types of cancer and the overexpression of EGFR in AdCC, it is reasonable to examine the correlation between angiogenesis and the EGFR signaling pathway in this carcinoma. In the present study, the expression of EGFR, CD31, CD146 and hypoxia-inducible factor-1α (HIF-1α) were evaluated by immunohistochemical staining with tissue microarray containing normal salivary gland (NSG), pleomorphic adenoma (PMA) and AdCC tissues. Pearson's correlation coefficient was conducted to demonstrate the correlation between EGFR, CD31, CD146 and HIF-1α. To determine their similarity and intimacy, hierarchical analysis was performed with Cluster 3.0 and then visualized using TreeView software. Immunohistochemical results of tissue microarrays were quantified, revealing that the expression of EGFR, CD146 and HIF-1α increased in AdCC compared with in PMA and NSG tissues. The association between the expression of EGFR and CD31 was significant and positive. The expression of CD146 and HIF-1α was positively correlated with EGFR and CD31, respectively. These findings suggest that the EGFR signaling pathway has a vital role in AdCC progression and may be associated with HIF-1α-mediated angiogenesis. These results may enhance our understanding of the mechanism underlying AdCC progression and provide potential clinical therapeutic strategies based on the inhibition of EGFR.

## Introduction

Adenoid cystic carcinoma (AdCC) is a rare major and minor salivary gland malignancy characterized by cellular and histopathological heterogeneity and a high incidence of distant metastasis in its early stage and local recurrence (1). With a high aggressive potential, AdCC is able to invade blood vessels and nerves even at an early stage (2). The current preventive and therapeutic methods for AdCC include chemotherapy, targeted agents and surgery. Unfortunately, these therapies only offer partial benefits and rarely improve the outcome (3). Therefore, novel therapeutic strategies are urgently required.

Epidermal growth factor receptor (EGFR) is a membrane-bound receptor. EGFR belongs to the ErbB family of receptors, which comprises EGFR (ErbB1), HER2/neu (ErbB2), ErbB3 and ErbB4 (4). EGFR is frequently overexpressed and mutated in various types of tumor, including lung cancer, oral squamous cell carcinoma and breast cancer (5,6). Anti-EGFR therapeutic approaches, specifically receptor blocking monoclonal antibodies and small molecule tyrosine kinase inhibitors, prolong tumor stabilization (7). EGFR-signaling pathways are also implicated in cell survival, proliferation, apoptotic resistance and invasion (8). Although EGFR has been extensively investigated in the context of AdCC, the association between EGFR and angiogenesis remains to be elucidated.

Angiogenesis is considered to be an important event during the progression of AdCC, due to the fact that blood vessels provide the nutrients to support tumor growth and also provide an entry site into the circulation for cancerous cells that have detached from the tumor mass, leading to distant metastasis (9). In contrast with normal vessels, vessels in tumors exhibit different characteristics. They are tortuous and dilated with excessive branching, shunts and have an uneven diameter (10). The walls of these tumor vessels are characterized by the absence of or discontinuous basement membrane and widened interendothelial junctions (11). With these defects, tumor vessels exhibit high vascular permeability and contribute to tumor metastasis (9). Generally, the adhesion molecule CD31 (platelet endothelial cell adhesion molecule) is used as a marker to indicate the presence of blood vessels. However, it has been reported that tumor vessels also highly express melanoma cell adhesion molecule (CD146), a member of the immunoglobulin gene superfamily (12). CD146 is expressed in >90% of cutaneous melanomas (13) and previous studies have demonstrated that the expression level of CD146 is associated with invasion and predicted metastatic potential of melanoma cells as well as AdCC (14,15). Genetic and pharmacological studies have also revealed that CD146 is implicated in important distinct aspects of biological processes in blood vessels and may correspond to endothelial permeability, therefore it may be used as a biomarker for pathological angiogenesis (16,17). Hypoxia-inducible factor-1α (HIF-1α), the main transcription factor involved in angiogenesis, was also analyzed in the present study (18,19).

In the present study, the expression of EGFR was determined in prospectively collected tumor tissues from a cohort of AdCC and pleomorphic adenoma (PMA) patients treated with surgery; the expression of EGFR was also detected in normal parotid glands. The association between CD146, HIF-1α, CD31 and EGFR was examined.

## Materials and methods

### Ethics statement

The present study was approved by the Medical Ethics Committee of the Hospital of Stomatology, Wuhan University (Wuhan, China) and was performed according to the the Declaration of Helsinki guidelines on experimentation involving human subjects. Written informed consent was obtained from participants.

### Patient samples and tissue microarray

A panel of AdCC and PMA at the Department of Oral and Maxillofacial Surgery, School and Hospital of Stomatology Wuhan University was identified by two independent pathologists according to the 2006 World Health Organization classification system (20). Tumor tissue microarrays were constructed in collaboration with Shanghai Biochip Co., Ltd. (Shanghai, China) and included 74 AdCC [cribriform pattern, 28; tubular pattern, 26; solid pattern, 20 as described previously (21)], 12 PMA and 18 normal salivary gland (NSG) tissues.

### Immunohistochemistry and scoring system

Immunohistochemistry was performed as previously described (22). Briefly, all slides were rehydrated and antigen retrieval was performed using sodium citrate (pH=6.0) in a pressure cooker with the exception of EGFR (EDTA buffer, pH=8.4). All slides were blocked with endogenous peroxidase with 3% hydrogen peroxide and blocked non-specific protein with 2.5% bovine serum albumin in phosphate-buffered saline. The following primary antibodies were used: Rabbit IgG EGFR (4267; 1:200; Cell Signaling Technology, Danvers, MA, USA), rabbit monoclonal HIF-1α, (ab190197; 1:200; Epitomics, Burlingame, CA, USA), rabbit polyclonal CD146 (17564-1-AP; 1:400; ProteinTech Group, Inc., Chicago, IL, USA), rabbit polyclonal CD31 (ab28364; 1:200; Epitomics) and slides were incubated at 4°C overnight with the diluted primary antibody. Slides were incubated with biotin-labeled secondary antibody [UltraSensitive™ S-P kit (mouse/rabbit); Fuzhou Maixin Biotechnology Co., Ltd., Fuzhou, China] and streptavidin peroxidase, visualized by 3,3′-diaminobenzidine and counterstained with hematoxylin. All slides were scanned using the Aperio ScanScope CS whole slice scanner (Aperio Technologies, Vista, CA, USA) with background substrate. The settings of the Aperio MVD algorithm were modified to allow identification of all CD31 stained blood vessels based on brown thresholds. The positive result was quantified using Aperio Quantification software (version 9.1; Aperio Technologies) for membrane, nuclear or pixel quantification and the membrane v9 algorithm was used to quantify the membranous expression of EGFR. Histoscores were calculated using the formula described previously (23).

### Hierarchical clustering and data visualization

Histoscores were converted into scaled values centered on zero in Microsoft Excel as described previously (22). Cluster 3.0 (http://bonsai.ims.u-tokyo.ac.jp/~mdehoon/software/cluster) with average linkage based on Pearson's correlation coefficient was used to achieve the hierarchical analysis and visualized via the Java TreeView 1.0.5 (http://jtreeview.sourceforge.net/).

### Statistical analysis

Data analysis was performed using GraphPad Prism 5.00 for Windows (GraphPad Software, Inc., La Jolla, CA, USA). One-way analysis of variance followed by Tukey's post-hoc test or Bonferroni multiple comparison was used to analyze the differences in immunohistochemical staining. The correlated expression of these markers was calculated using two-tailed Pearson's correlation following confirmation of the sample with Gaussian distribution. All values are expressed as the mean ± standard error of the mean. P<0.05 was considered to indicate a statistically significant difference.

## Results

### Association between the expression of EGFR and CD31 in NSG, PMA and AdCC tissues

**Figure 1:**
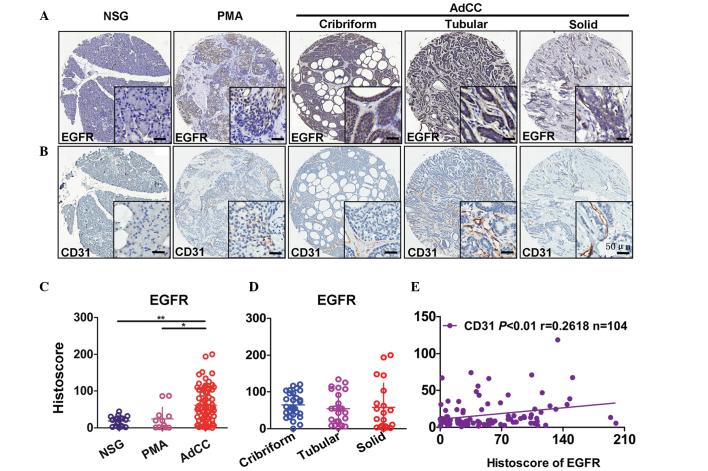
Association between the expression of EGFR and CD31 in NSG, PMA and AdCC tissues. Representative immunohistochemical staining of (A) EGFR membranous expression and (B) CD31 membranous expression in human NSG, PMA and cribriform, tubular or solid type AdCC tissues. Scale bar=50 *µ*m. Quantification of EGFR expression levels in (C) human NSG, PMA and AdCC tissues and (D) subtypes of AdCC using an AperioScanscope scanner and software. Data were analyzed by Graph Pad Prism 5 software. Data are presented as the mean ± standard error of the mean. ^*^P<0.05, AdCC vs. PMA tissues; ^**^P<0.01, AdCC vs. NSG tissues. (E) Correlation between EGFR and CD31 expression levels in human NSG, PMA and AdCC tissues (P<0.01, r=0.2618, n=104) using two-tailed Pearson's test. EGFR, epidermal growth factor receptor; NSG, normal salivary gland; PMA, polymorphism adenoma; AdCC, adenoid cystic carcinoma.

The expression of EGFR was initially evaluated by immunohistochemical staining. Representative immunostained EGFRs in NSG, PMA and AdCC are shown in Fig. 1A. The expression of EGFR was increased in AdCC tissues compared with NSG and PMA tissues. EGFR-positive cases presented a membranous pattern in NSG tissues. By contrast, EGFR-positive samples in PMA and AdCC tissues demonstrated a mixed cell cytoplasmic and membranous pattern. Additionally, EGFR was strongly expressed in the edges of the cancer nests in the three subtypes of AdCC (Fig. 1A). As shown in Fig. 1B, the staining intensity of CD31 was increased in NSG, PMA and AdCC tissues. By calculating weak positive and strong positive cases as positive, 63 out of 74 AdCC, 3 out of 12 PMA and 3 out of 18 NSG tissues were found to be EGFR positive (Fig. 1C). Among the subtype groups of AdCC, the difference in expression of EGFR was not significant. Notably, the staining of EGFR in cribriform and tubular forms was evidently stronger than that in solid forms (Fig. 1D). To determine the association between EGFR and angiogenesis, CD31 was subjected to immunohistochemical staining. To verify whether or not EGFR is involved in AdCC angiogenesis, the two-tailed Pearson's correlation was conducted and immunohistochemical staining scores were analyzed. The levels of EGFR were positively correlated with the expression of CD31 (P<0.01, r=0.2618, n=104), suggesting a possible role of the EGFR-signaling pathway in AdCC angiogenesis.

### Expression of HIF-1α and CD146 in NSG, PMA and AdCC tissues

**Figure 2:**
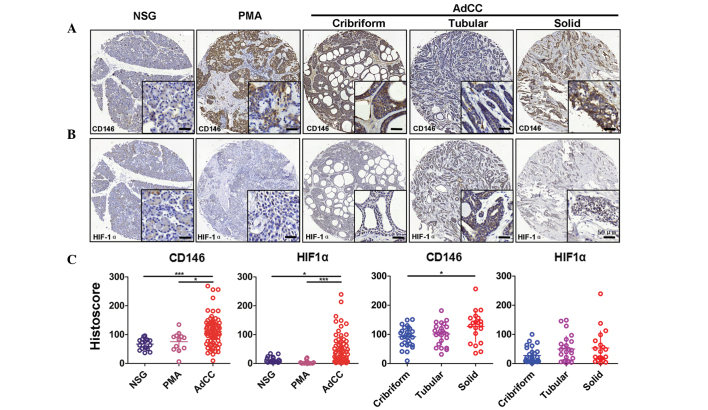
Expression of HIF-1α and CD146 in NSG, PMA and AdCC tissues. Representative immunohistochemical staining of (A) CD146 membranous expression and (B) HIF-lα cytoplasmic and nuclear expression in human NSG, PMA and cribriform, tubular or solid type AdCC tissues. Scale bar=50 *µ*m. (C) Quantification of HIF-1α and CD146 expression levels in human NSG, PMA and AdCC tissues and subtypes of AdCC using an AperioScanscope scanner and software. Data were analyzed using Graph Pad Prism 5 software. Data are expressed as the mean ± standard error of the mean. ^*^P<0.05, AdCC vs. PMA tissues in CD146, AdCC vs. NSG tissues in HIF1α, solid vs. cribriform subtype tissues in CD146; ^***^P<0.001, AdCC vs. NSG tissues in CD146, AdCC vs. PMA tissues in HIF1α. EGFR, epidermal growth factor receptor; NSG, normal salivary gland; PMA, polymorphism adenoma; AdCC, adenoid cystic carcinoma; HIF-1α, hypoxia-inducible factor-1α.

Considering the significant role of HIF-1α in angiogenesis in various types of cancer and the pathological angiogenesis biomarker, CD146, in tumor-derived angiogenesis, HIF-1α and CD146 immunostaining was performed to examine the expression of CD146 and HIF-1α in AdCC, PMA and NSG tissues. The results demonstrated that CD146 was expressed at the membrane in NSG, PMA and AdCC tissues. CD146 was strongly stained at the membrane in AdCC tissues, which were distributed in the interstitial tissues and were also highly expressed in the inner epithelial ductal cells of tubular pattern and irregular cancer nests of cribriform form (Fig. 2A). In the NSG tissue, HIF-1α staining was present in the cytoplasm and the nucleus of the cell, whereas, in PMA and AdCC tissues, HIF-1α staining was largely restricted to the nuclear area (Fig. 2B). Notably, the levels of CD146 in the solid form of AdCC were significantly higher than those in the cribriform subtype. However, the difference between the mean levels of CD146 in the tubular subtype and the two other subtypes of AdCC was not significant (Fig. 2C). By quantifying immunohistochemical staining, the results demonstrated that the expression of HIF-1α and CD146 was significantly increased in AdCC (Fig. 2C) compared with NSG and PMA tissues. The results also suggested that 42 out of 74 were HIF-1α positive and 46 out of 74 were CD146 positive. In NSG tissues, 6 out of 18 were HIF-1α positive and 4 out of 18 were CD146 positive. In PMA tissues, 3 out of 12 were HIF-1α positive and 5 out of 12 were CD146 positive (Fig. 2C). Hypoxia is a widespread phenomenon in the three subtypes of AdCC as HIF-1α staining demonstrated no significance in all of the three subtypes (Fig. 2C).

### Close correlations among HIF-1α, CD146 and EGFR in AdCC

**Figure 3:**
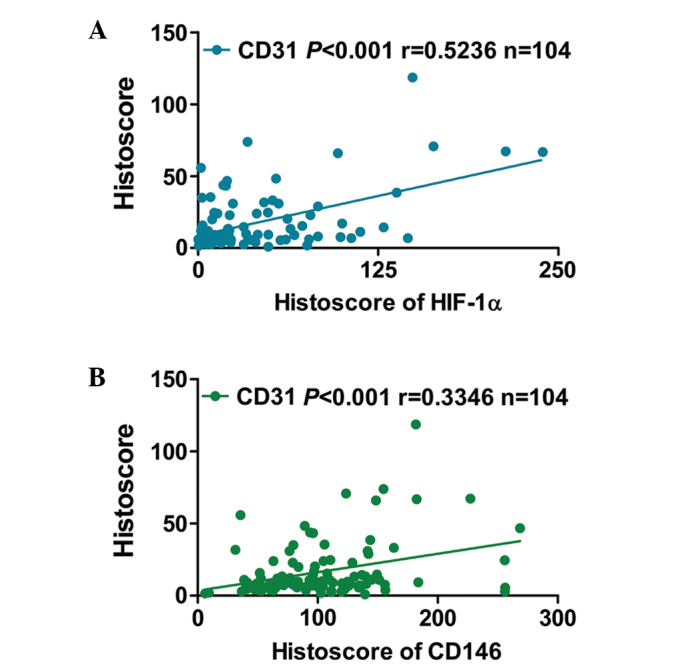
HIF-1α and CD146 may be involved in angiogenesis in AdCC. Correlation and regression of HIF-1α and CD146 in human NSG, PMA and AdCC tissues. (A) Correlation between HIF-1α with CD31 expression levels in human NSG, PMA and AdCC tissues (P<0.001, r=0.5236, n=104). (B) Correlation between CD146 with CD31 expression levels in human NSG, PMA and AdCC tissues (P<0.001, r=0.3346, n=104) using two-tailed Pearson's test. HIF-1α, hypoxia-inducible factor-1α; NSG, normal salivary gland; PMA, polymorphism adenoma; AdCC, adenoid cystic carcinoma.

**Figure 4:**
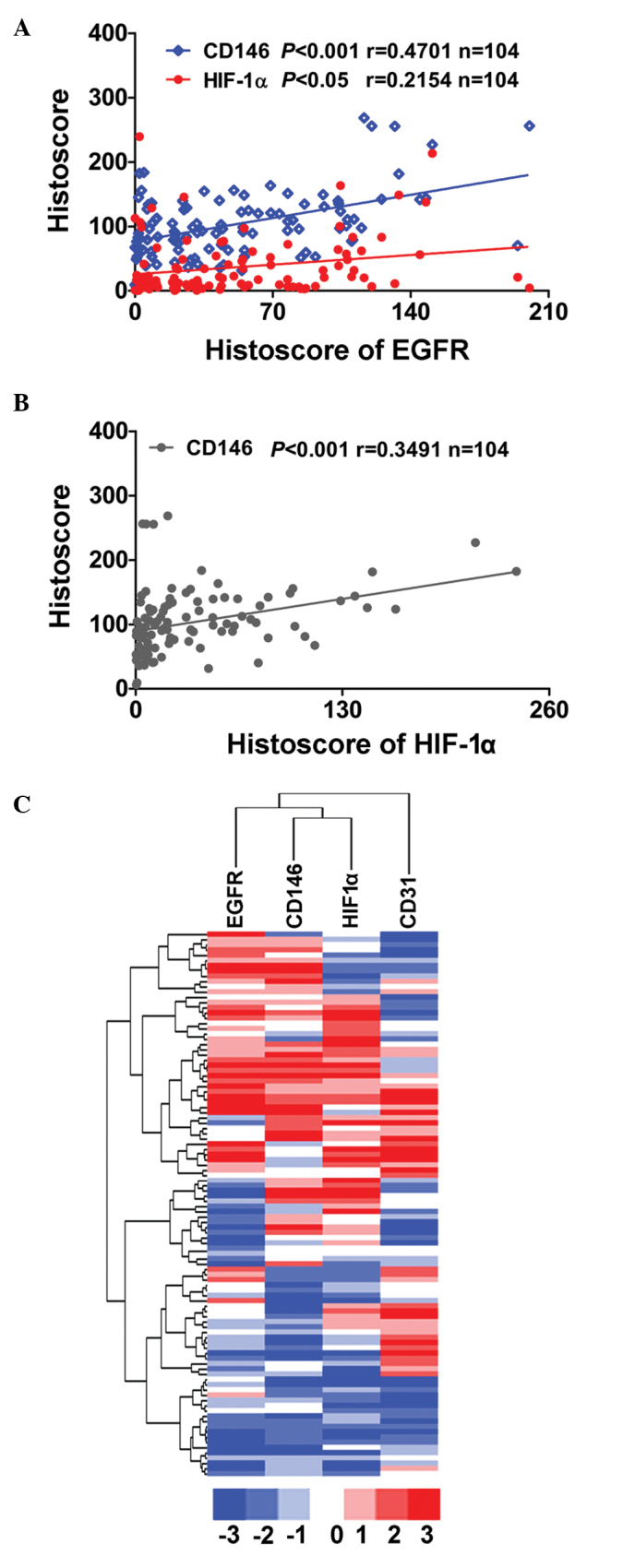
Overexpression of EGFR is correlated with HIF-1α and CD146 in human AdCC tissue. (A) Expression of EGFR was positively correlated with HIF1-α and CD146 (P<0.05, r=0.2154, n=104; P<0.001, r=0.4701, n=104, respectively) and the (B) expression of HIF-1α was significantly correlated with CD146 (P<0.001, r=0.3491, n=104) in human NSG, PMA and AdCC tissues by analyzing the tissue microarray immunohistochemical staining. (C) Hierarchical clustering of immunohistochemical results of human AdCC with EGFR, HIF-1α and CD146 (statistics including AdCC tissue only n=74). EGFR, epidermal growth factor receptor; NSG, normal salivary gland; PMA, polymorphism adenoma; AdCC, adenoid cystic carcinoma; HIF-1α, hypoxia-inducible factor-1α.

Since EGFR and CD31 were demonstrated to be significantly correlated with each other, the correlation between the expression of CD31, HIF-1α, CD146 and EGFR was measured in AdCC tissues, in which the two-tailed Pearson's correlation was performed. Pearson's correlation of cases with interpretable scores of CD31 and HIF-1α (P<0.001, r=0.5236, n=104) demonstrated a positive correlation (Fig. 3A). Similar results were also observed with CD31 and CD146 (Fig. 3B; P<0.001, r=0.3346, n=104). These results suggested that HIF-1α and CD146 may be involved in the angiogenesis of AdCC. To verify the association between HIF-1α and CD146 with EGFR in AdCC, the present study then performed a correlation analysis between HIF-1α and CD146 with EGFR. The levels of HIF-1α and CD146 were positively correlated with the expression of EGFR (P<0.05, r=0.2154, n=104, P<0.001, r=0.4701, n=104, respectively; Fig. 4A). The expression of HIF-1α was positively correlated with that of CD146 (Fig. 4B; P<0.001, r=0.3491, n=104). These associations between angiogenic factors in human AdCC were displayed in a visual image (Fig. 4C), which was obtained by hierarchical clustering.

## Discussion

Angiogenesis is a fundamental event in various physiological and pathological processes, including embryo implantation the menstrual cycle, rheumatic disease and cancer (9). Tumor angiogenesis is a key mechanism for tumor growth and metastasis. Hence, targeting angiogenesis has been regarded as a promising therapy for cancer and other angiogenesis-associated diseases (9). Although agents that inhibit tumor angiogenesis have been applied, the regulatory mechanisms of angiogenesis remain to be elucidated (24). In the present study, the important role of EGFR in the angiogenesis of human AdCC was uncovered by immunohistochemical analysis. The results suggested that EGFR was highly expressed in human salivary gland AdCC tissues compared with in PMA and NSG tissues. In addition, EGFR levels were positively correlated with the expression of HIF-1α, CD146 and CD31 in human AdCC. These results revealed that EGFR is possibly involved in angiogenesis by affecting the expression of HIF-1α and CD146 in human AdCC.

In the present study, CD31 and CD146 were used as angiogenic markers in order to analyze the blood vessel distribution and status in this carcinoma. A higher expression of CD31 and CD146 was identified in the AdCC tissues compared with in the PMA and NSG tissues. CD31 is a common endothelial cell marker that is widely used to monitor blood vessels, while CD146 is a structural component of interendothelial junctions and is particularly highly expressed in pathological vessels (25). It was reported that CD146 could bind to vascular endothelial growth factor receptor (VEGFR)-2 and mediate the phosphorylation of VEGFR-2, as well as the downstream signaling pathways Akt/p38 and MAPK/NF-κB, which promote endothelial cell migration and microvascular formation in tumors, therefore promoting the development of tumors (26). Additionally, CD146 positive endothelial cells were considered to be fully undifferentiated, revealing distinguished characteristics compared with differentiated endothelial cells (17). Therefore, CD146 was selected as a tumor-associated endothelial biomarker in the present study. CD146 positive cells were found in the interstitial tissues of AdCC, suggesting the formation of pathological blood vessels in human AdCC. Notably, strong staining of CD146 was identified in the inner epithelial ductal cells of tubular pattern and irregular cancer nests of cribriform form. Since CD146 was found to be highly expressed in cutaneous melanoma, a severe type of malignant cancer due to its high rate of distant metastasis, CD146 blockade may inhibit tumor growth and metastasis of human melanoma (27). Therefore, the high expression of CD146 in AdCC tumor cells may explain the invasive tendency of AdCC and early hematogenous metastasis. Currently, CD146 targeting therapies have been proposed. It was reported that treatment with microRNA-329 in a mouse model decreased excessive CD146 expression in blood vessels, which could significantly repress the neovascularization of tumors and therefore attenuate tumor growth (28). Furthermore, anti-CD146 monoclonal antibody was demonstrated to inhibit angiogenesis by suppressing NF-κB activation (29). By examining the association between EGFR and CD146 in AdCC, the present study found that EGFR was significantly correlated with CD146, suggesting CD146 may be regulated by EGFR. However, this requires further investigation in the future.

By contrast, a significant correlation was identified between HIF-1α and CD146, as well as HIF-1α and EGFR. Hypoxia was considered to be the most important microenvironment in tumor development (30). By regulating the transcription of angiogenic factors, including VEGF, basic fibroblast growth factor and matrix metalloproteinase-9, HIF-1α could promote tumor-derived angiogenesis (31). In the present study, the positive nuclear immunoreactivity staining of HIF-1α was distributed in all three forms of AdCC. In addition, the simultaneous high EGFR levels and HIF-1α expression were observed in numerous cases of AdCC and the correlation analysis suggested a significant association between EGFR with HIF-1α. These data indicated that EGFR may have potentially contributed to HIF-1α nuclear translocation as previously reported (32). Cetuximab, an anti-EGFR antibody used for cancer therapy, was reported to be able to sensitize human head and neck squamous cell carcinoma cells to radiation in part through inhibiting radiation-induced upregulation of HIF-1α (33). In addition, HIF-1α was verified to be required in HER2/neu (ERBB2)-mediated mammary tumor growth and anoikis resistance (34). This suggested that the EGFR signaling pathway acts as an upstream regulator for HIF-1α and that targeting EGFR may be beneficial for cancer treatment by affecting HIF-1α expression and translocation. The ERBB-receptor network exemplifies the pathogenic ability of aberrations in biological information transfer and is considered as one of the most extensively investigated areas of signal transduction (35). EGFR reportedly exhibits positive AdCCs (67–85%) (36,37), in accordance with the results of the present study. Another study in our laboratory suggested that targeting EGFR could repress the invasion and distant metastasis of human AdCC via downregulating epithelial-mesenchymal transition and related anoikis resistance (38). In addition, as discussed previously, targeting EGFR could be beneficial for cancer therapy by repressing cell growth, as well as inducing the apoptosis of cancer cells. The results of the present study suggest that targeting EGFR may suppress HIF-1α-mediated tumor angiogenesis. In addition, considering the remediability of EGFR, indicating that EGFR could be targeted by monoclonal antibodies or small therapeutic molecules, including cetuximab and gefitinib and therefore treat cancer, it may be a potential option for the treatment of AdCC.

In conclusion, the present study verified that the expression of EGFR was highly correlated with CD31, CD146 and HIF-1α in human AdCC and also suggested a possible association between EGFR and tumor-derived angiogenesis in this tumor. Thus, targeting EGFR may provide a possible novel therapeutic strategy for the treatment of human AdCC.
