# [Corrigendum] Role of hypoxia-inducible factor-1α and CD146 in epidermal growth factor receptor-mediated angiogenesis in salivary gland adenoid cystic carcinom

**DOI:** 10.3892/mmr.2026.13801

**Published:** 2026-01-13

**Authors:** Wei-Ming Wang, Zhi-Li Zhao, Wen-Feng Zhang, Yi-Fang Zhao, Lu Zhang, Zhi-Jun Sun

Mol Med Rep 12: 3432–3438, 2015; DOI: 10.3892/mmr.2015.3815

Following the publication of the above paper, the authors contacted the Editor to explain that they had made a couple of inadvertent errors in assembling the data in Figs. 1B and 2B. Specifically, the following issues were identified: first, the immunohistochemical staining images representing CD31 in Fig. 1B on p. 3434 were chosen from the wrong dataset; secondly, the immunohistochemical staining images representing HIF-1α in Fig. 2B on p. 3435 were similarly included in this figure incorrectly. After having performed an independent analysis of these data in the Editorial Office, it came to light that certain of the data featured in Fig. 2B had been submitted for publication at around the same time in an article featuring some of the same authors to the journal *PLoS One*.

However, the authors were able to consult their original data, and the revised versions of Figs. 1 and 2, now featuring all the correct data for Figs. 1B and 2B, are shown on the next two pages. Note that these errors did not adversely affect either the results or the overall conclusions reported in this study. All the authors agree with the publication of this corrigendum, and are grateful to the Editor of *Molecular Medicine Reports* for allowing them the opportunity to publish this. They also wish to apologize to the readership of the Journal for any inconvenience caused.

## Figures and Tables

**Figure 1.**
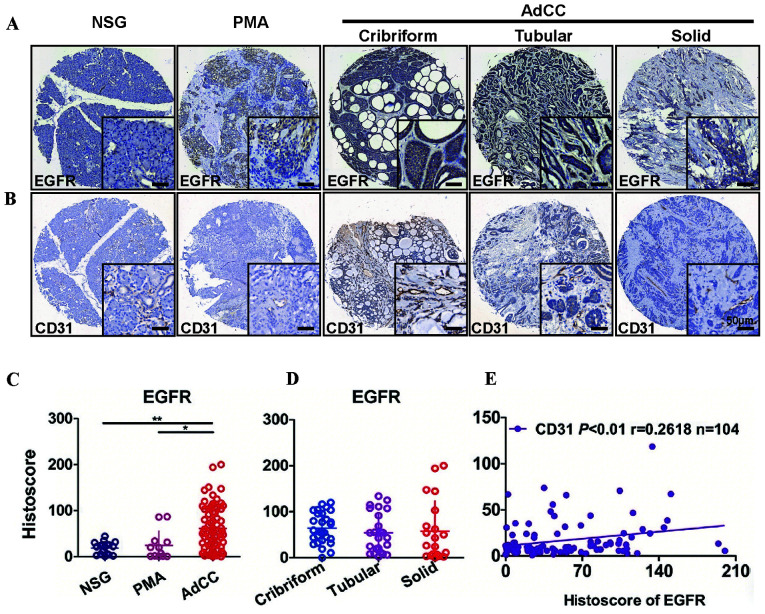
Association between the expression of EGFR and CD31 in NSG, PMA and AdCC tissues. Representative immunohistochemical staining of (A) EGFR membranous expression and (B) CD31 membranous expression in human NSG, PMA and cribriform, tubular or solid type AdCC tissues. Scale bar=50 µm. Quantification of EGFR expression levels in (C) human NSG, PMA and AdCC tissues and (D) subtypes of AdCC using an AperioScanscope scanner and software. Data were analyzed by Graph Pad Prism 5 software. Data are presented as the mean ± standard error of the mean. *P<0.05, AdCC vs. PMA tissues; **P<0.01, AdCC vs. NSG tissues. (E) Correlation between EGFR and CD31 expression levels in human NSG, PMA and AdCC tissues (P<0.01, r=0.2618, n=104) using two-tailed Pearson's test. EGFR, epidermal growth factor receptor; NSG, normal salivary gland; PMA, polymorphism adenoma; AdCC, adenoid cystic carcinoma.

**Figure 2.**
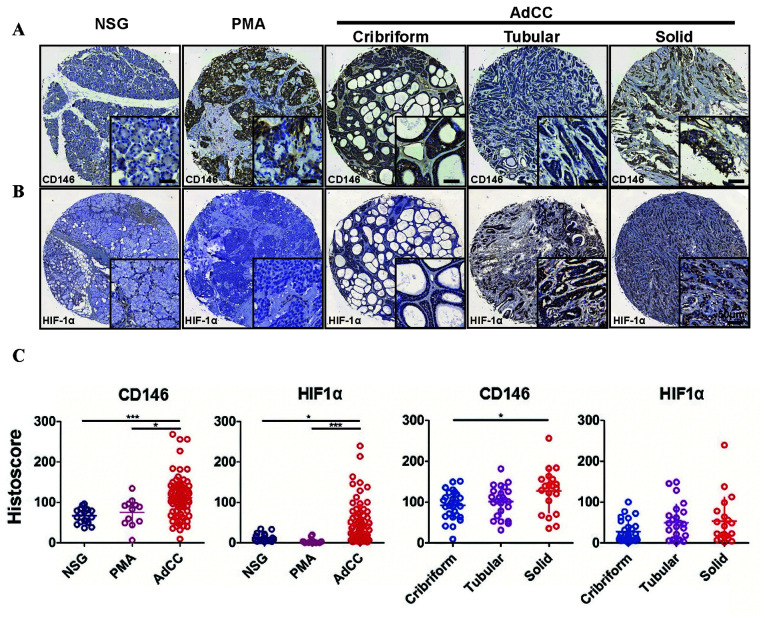
Expression of HIF-1α and CD146 in NSG, PMA and AdCC tissues. Representative immunohistochemical staining of (A) CD146 membranous expression and (B) HIF-1α cytoplasmic and nuclear expression in human NSG, PMA and cribriform, tubular or solid type AdCC tissues. Scale bar=50 µm. (C) Quantification of HIF-1α and CD146 expression levels in human NSG, PMA and AdCC tissues and subtypes of AdCC using an AperioScanscope scanner and software. Data were analyzed using Graph Pad Prism 5 software. Data are expressed as the mean ± standard error of the mean. *P<0.05, AdCC vs. PMA tissues in CD146, AdCC vs. NSG tissues in HIF-1α, solid vs. cribriform subtype tissues in CD146; ***P<0.001, AdCC vs. NSG tissues in CD146, AdCC vs. PMA tissues in HIF-1α. EGFR, epidermal growth factor receptor; NSG, normal salivary gland; PMA, polymorphism adenoma; AdCC, adenoid cystic carcinoma; HIF-1α, hypoxia-inducible factor-1α.

